# Prognosis Prediction of Measurable Enhancing Lesion after Completion of Standard Concomitant Chemoradiotherapy and Adjuvant Temozolomide in Glioblastoma Patients: Application of Dynamic Susceptibility Contrast Perfusion and Diffusion-Weighted Imaging

**DOI:** 10.1371/journal.pone.0113587

**Published:** 2014-11-24

**Authors:** Jae Hyun Kim, Seung Hong Choi, Inseon Ryoo, Tae Jin Yun, Tae Min Kim, Se-Hoon Lee, Chul-Kee Park, Ji-Hoon Kim, Chul-Ho Sohn, Sung-Hye Park, Il Han Kim

**Affiliations:** 1 Department of Radiology, Seoul National University College of Medicine, Seoul, Korea; 2 Center for Nanoparticle Research, Institute for Basic Science, and School of Chemical and Biological Engineering, Seoul National University, Seoul, Korea; 3 Department of Internal Medicine, Cancer Research Institute, Seoul National University College of Medicine, Seoul, Korea; 4 Department of Neurosurgery, Seoul National University College of Medicine, Seoul, Korea; 5 Department of Pathology, Seoul National University College of Medicine, Seoul, Korea; 6 Department of Radiation Oncology, Cancer Research Institute, Seoul National University College of Medicine, Seoul, Korea; The Ohio State University Medical Center, United States of America

## Abstract

**Purpose:**

To assess the prognosis predictability of a measurable enhancing lesion using histogram parameters produced by the normalized cerebral blood volume (nCBV) and normalized apparent diffusion coefficient (nADC) after completion of standard concomitant chemoradiotherapy (CCRT) and adjuvant temozolomide (TMZ) medication in glioblastoma multiforme (GBM) patients.

**Materials and Methods:**

This study was approved by the institutional review board (IRB), and the requirement for informed consent was waived. A total of 59 patients with newly diagnosed GBM who received standard CCRT with TMZ and adjuvant TMZ for six cycles underwent perfusion-weighted and diffusion-weighted imaging. Twenty-seven patients had a measurable enhancing lesion and 32 patients lacked a measurable enhancing lesion based on the Response Assessment in Neuro-Oncology (RANO) criteria in the follow-up MRI, which was performed within 3 months after adjuvant TMZ therapy was completed. We measured the nCBV and nADC histogram parameters based on the measurable enhancing lesion. The progression free survival (PFS) was analyzed by the Kaplan-Meier method with the use of the log-rank test.

**Results:**

The median PFS of patients lacking measurable enhancing lesion was longer than for those with measurable enhancing lesions (17.6 vs 3.3 months, *P*<.0001). There was a significant, positive correlation between the 99^th^ percentile nCBV value of a measurable enhancing lesion and the PFS (*P* = .044, R^2^ = .152). In addition, the median PFS was longer in patients with a 99^th^ percentile nCBV value ≧4.5 than it was in those with a value <4.5 (4.4 vs 3.1 months, *P* = .036).

**Conclusion:**

We found that the nCBV value can be used for the prognosis prediction of a measurable enhancing lesion after the completion of standard treatment for GBM, wherein a high 99^th^ percentile nCBV value (≧4.5) suggests a better PFS for GBM patients.

## Introduction

Glioblastoma multiforme (GBM) is the most common primary brain tumor in adults; it is also extremely aggressive. In spite of enormous treatment efforts, the prognosis is grave, with the median survival rate ranging from 9 to 18 months [1]–[4]. The standard treatment for newly diagnosed GBM consists of maximal surgical resection and concurrent chemoradiotherapy (CCRT) with temozolomide (TMZ), followed by 6 cycles of adjuvant TMZ [5]–[7]. The radiologic assessment, especially via magnetic resonance imaging (MRI), plays an important role in the evaluation of the GBM response to treatment. In 1990, Macdonald introduced radiological and clinical response criteria for malignant brain tumors [8]. These criteria provide a standardized radiological assessment of the tumor response and are based on measuring the enhancing component of the tumor. The enhancing portion of GBM is a key factor for using these criteria to predict the prognosis of GBM patients. Furthermore, recently, the Response Assessment in Neuro-Oncology (RANO) Working Group proposed new standardized criteria for accurately assessing the tumor response in high-grade glioma patients [9]. The RANO criteria emphasize not only the evaluation of the non-enhancing component but also precise examination of measurable enhancing tumor components. The measurable enhancing lesions are defined as bidimensionally contrast-enhancing lesions with clearly defined margins by computed tomography (CT) or MRI scans and two perpendicular diameters of at least 10 mm visible on two or more axial slices that are preferably, at most, 5 mm apart with 0-mm skip [9]. The presence of a measurable enhancing lesion is an important requirement for defining GBM progression with the RANO criteria.

Many researchers have tried to predict the prognosis of patients with high-grade glioma with advanced MR imaging techniques, such as dynamic susceptibility contrast (DSC) perfusion-weighted imaging (PWI) and diffusion-weighted imaging (DWI) [10]–[16]. Although the measurable enhancing lesion is a significant factor for assessing the tumor response in GBM patients, no clinical studies have evaluated the impact of the presence of a measurable enhancing lesion after the completion of standard treatment combined with adjuvant TMZ. Therefore, the purpose of this study was to assess the prognosis predictability in GBM patients of a measurable enhancing lesion after the completion of standard CCRT and adjuvant TMZ using histogram parameters produced by DSC-PWI and DWI; several studies have shown that histogram analysis of these advanced MR imaging methods is useful in predicting early treatment response or prognosis in patients with high-grade glioma [17]–[21].

## Materials and Methods

Our study was approved by the Institutional Review Board (IRB) at Seoul National University Hospital (IRB No. H-1401-073-550). The institutional review board waived the need for written informed consent from the participants because this was a retrospective study and the data were analyzed anonymously.

### Patients

**Figure 1:**
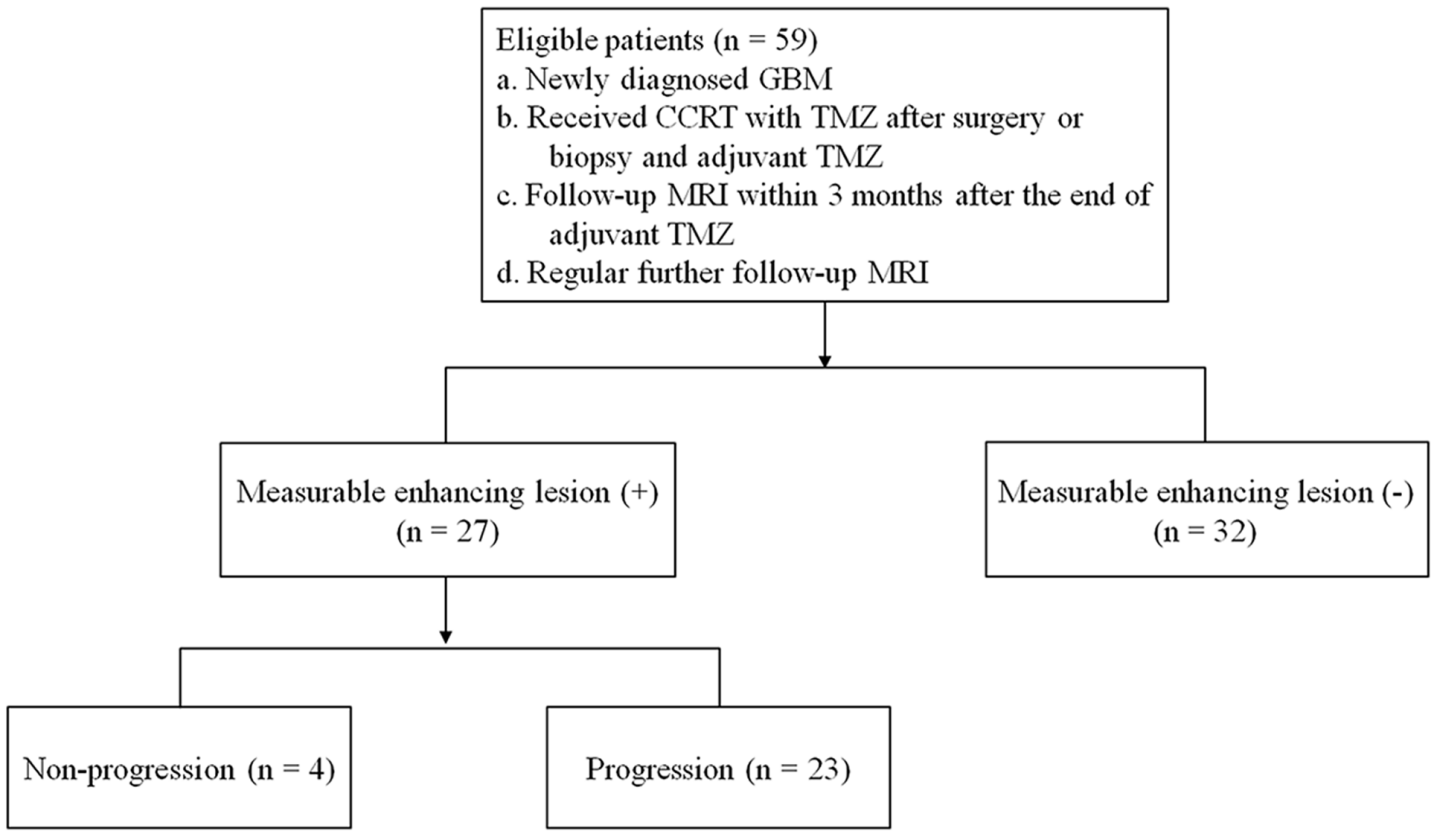
Flow diagram of the patients. GBM = glioblastoma multiforme, CCRT = concurrent chemoradiotherapy, TMZ = temozolomide.

From November 2006 to June 2013, 338 patients with newly diagnosed GBM who had undergone surgical resection or stereotactic biopsy were selected. The inclusion criteria were as follows: the patient (a) had a histopathologic diagnosis of GBM based on the World Health Organization criteria; (b) underwent standard CCRT with TMZ after surgery or biopsy and 6 cycles of adjuvant TMZ; (c) had undergone a follow-up MRI study within three months (mean duration: 25 days, range: 0–84 days) of completing 6 cycles of adjuvant TMZ, which included DWI and DSC-PWI sequences; and (d) had also undergone additional regular follow-up MRI studies. Finally, 59 GBM patients (42 male, 17 female; age range, 12–81 years; mean age, 50 years) were enrolled in the present study. We divided these 59 patients into two groups. One group had measurable enhancing lesions, which are defined as bidimensionally contrast-enhancing lesions with clearly defined margins visible with MRI and two perpendicular diameters of at least 10 mm visible on two or more axial slices that are preferably, at most, 5 mm apart with 0-mm skip [9], on the first follow-up MRI study (*n* = 27; 18 male, 9 female; age range, 16–68 years; mean age, 54 years). The other group lacked measurable enhancing lesions (*n* = 32; 24 male, 8 female; age range, 12–81 years; mean age, 48 years). We divided the 27 patients with measurable enhancing lesion into the following two subgroups: the non-progression group, which is defined as the patients who did not show disease progression within the entire follow-up period (*n* = 4), and the progression group (*n* = 23) (Fig. 1).

### Image Acquisition

For each patient, the first follow-up MRI after the end of 6 cycles of adjuvant TMZ was performed using 1.5 T scanners [*n* = 22, Signa Excite 1.5T (*n* = 7); Signa HDxt 1.5T (*n* = 15), GE Medical Systems, Milwaukee, WI, USA] or 3 T scanners [*n* = 37, Verio (*n* = 28); Trio Tim (*n* = 2), Siemens Medical Solution, Erlangen, Germany; Signa Excite 3.0T (*n* = 6), Signa HDxt 3.0T (*n* = 1), GE Medical Systems, Milwaukee, WI, USA]. The imaging sequences for the brain included axial spin-echo T1-weighted (T1W) images, fast/turbo spin-echo T2-weighted (T2W) images, fluid-attenuated inversion-recovery (FLAIR) images, DWI, DSC-PWI with gadobutrol (Gadovist, Bayer Schering Pharma, Berlin, Germany) and subsequent contrast-enhanced spin-echo T1W images. The MR imaging parameters were as follows: 558–650/8–20 ms/70–90°/384×192–212 (TR/TE/FA/matrix) for spin-echo T1W images; 4500–5160/91–106.3 ms/90–130°/448–640×220–348 (TR/TE/FA/matrix) for fast spin-echo T2W images; and 9000–9900/97–162.9 ms/90–130°/199–384×209–220 (TR/TE/FA/matrix) for FLAIR images. The other parameters for the three images were as follows: section thickness of 5 mm with a 1 mm gap and a field of view (FOV) of 199–240×199–240 mm.

DWI was performed with a single-shot, spin-echo EPI sequence in the axial plane before the injection of contrast material with a TR/TE of 6900–10000/55–70 ms at b = 0 and 1000 sec/mm^2^, 25–40 sections, a 3–4 mm section thickness, a 1 mm intersection gap, a FOV of 220–240×220–240 mm, a matrix of 128–240×128–192, three signal averages, and a voxel resolution of 1.5×1.5×3.0–4.0 mm. DWI was acquired in three orthogonal directions and combined into a trace image. Using these data, ADC maps were calculated on a voxel-by-voxel basis with the software incorporated into the MRI unit.

For DSC-PWI, a single-shot gradient-echo EPI sequence was used during the intravenous injection of contrast agent. The DSC PWI imaging parameters were as follows: TR/TE, 1500/30–40 ms; FA, 35–90°; FOV, 220–240×220–240 mm; 15–20 sections; matrix, 128×128; section thickness, 5–6 mm; intersection gap, 1–1.5 mm; and voxel resolution, 1.86×1.86×5 mm. For each section, 60 images were obtained at intervals equal to the repetition time. After four to five time points, a bolus of gadobutrol at a dose of 0.1 mmol/kg of body weight and a rate of 4 mL/sec was injected with an MR-compatible power injector (Spectris; Medrad, Pittsburgh, PA, USA). The bolus of contrast material was followed by a 30 mL bolus of saline, which was administered at the same injection rate.

### Follow-up and Progression Assessment

The 59 patients were followed up during a median period of 10 months (range, 1.4–47.8 months). Clinical features and follow-up MRI were used to assess the patients. We evaluated the disease progression and compared it with the imaging features and clinical status of the patients at the time of the first follow-up MRI after adjuvant TMZ. The determination of the disease progression was based on the RANO criteria [9]. The patients who met any one of following criteria were classified as having progressive disease: (a) ≧25% increase in the sum of the products of the perpendicular diameters of enhancing lesions with the smallest tumor measurement; (b) any new lesion; (c) clear clinical deterioration not attributable to other causes apart from the tumor; (d) failure to return for evaluation as a result of death or deteriorating condition; and (e) clear progression of nonmeasurable disease. We also evaluated the pseudoprogression based on the RANO criteria [9] in the measurable enhancing lesion (+) group (*n* = 27), because there was a possibility that the pseudoprogression influenced the normalized CBV (nCBV) of the measurable enhancing lesion. One patient of the progression group was not evaluated due to lack of follow-up MRI within 12 weeks after the radiation treatment. One radiologist (S.H.C.; 12 years of brain MRI experience) reviewed all follow-up MR images obtained from the study population (*n* = 59).

### Quantitative Image Analysis

The MR data for the ADC_1000_ and the DSC-PWI of the patients with measurable enhancing lesions (*n* = 27) were digitally transferred from the picture archiving and communication system workstation to a personal computer for further analysis. Relative CBV (rCBV) maps were obtained by using a dedicated software package (NordicICE and TumorEx; NordicNeuroLab, Bergen, Norway) with an established tracer kinetic model applied to the first-pass data [22], [23]. First, realignment was performed to minimize patient motion during the dynamic scanning. The gamma-variate function, which is an approximation of the first-pass response as it would appear in the absence of recirculation, was fitted to the 1/T2^*^ curves to reduce the effects of recirculation. The dynamic curves were mathematically corrected to reduce contrast agent leakage effects [24]. After eliminating recirculation and contrast agent leakage, the rCBV was computed with numeric integration of the curve. To minimize variances in the rCBV value in an individual patient, the pixel-based rCBV maps were normalized by dividing every rCBV value in a specific section by the rCBV value in the unaffected white matter as defined by a neuroradiologist (S.H.C.) [25]. Coregistration between the contrast-enhanced T1W images and the nCBV maps and between the contrast-enhanced T1W images and the ADC maps was performed based on geometric information stored in the respective data sets with the use of a dedicated software package (NordicICE) [26]. The differences in the slice thickness between images were corrected automatically by re-slicing and coregistration, which was based on the underlay and structural images. The nCBV and ADC maps were displayed as color overlays on the contrast-enhanced T1W images. The regions of interest (ROIs) for the measurable enhancing lesion in each section of the contrast-enhanced T1W images were determined by the semiautomatic segmentation method using dedicated software (Nordic TumorEx), in which the contrast-enhanced T1W images were used for the structural images [27]. The ROI volumes were also automatically calculated from the ROIs determined by the semiautomatic segmentation method.

To minimize the bias from the use of multiple MR scanners, we used the normalized ADC (nADC) value to define the foci with diffusion restriction in the measurable enhancing lesion [28]. The nADC value of each voxel was defined as the ADC value of the voxel divided by the ADC value of normal periventricular white matter. The ADC values of the normal periventricular white matter were measured at the contralateral side of the main tumor.

**Figure 2:**
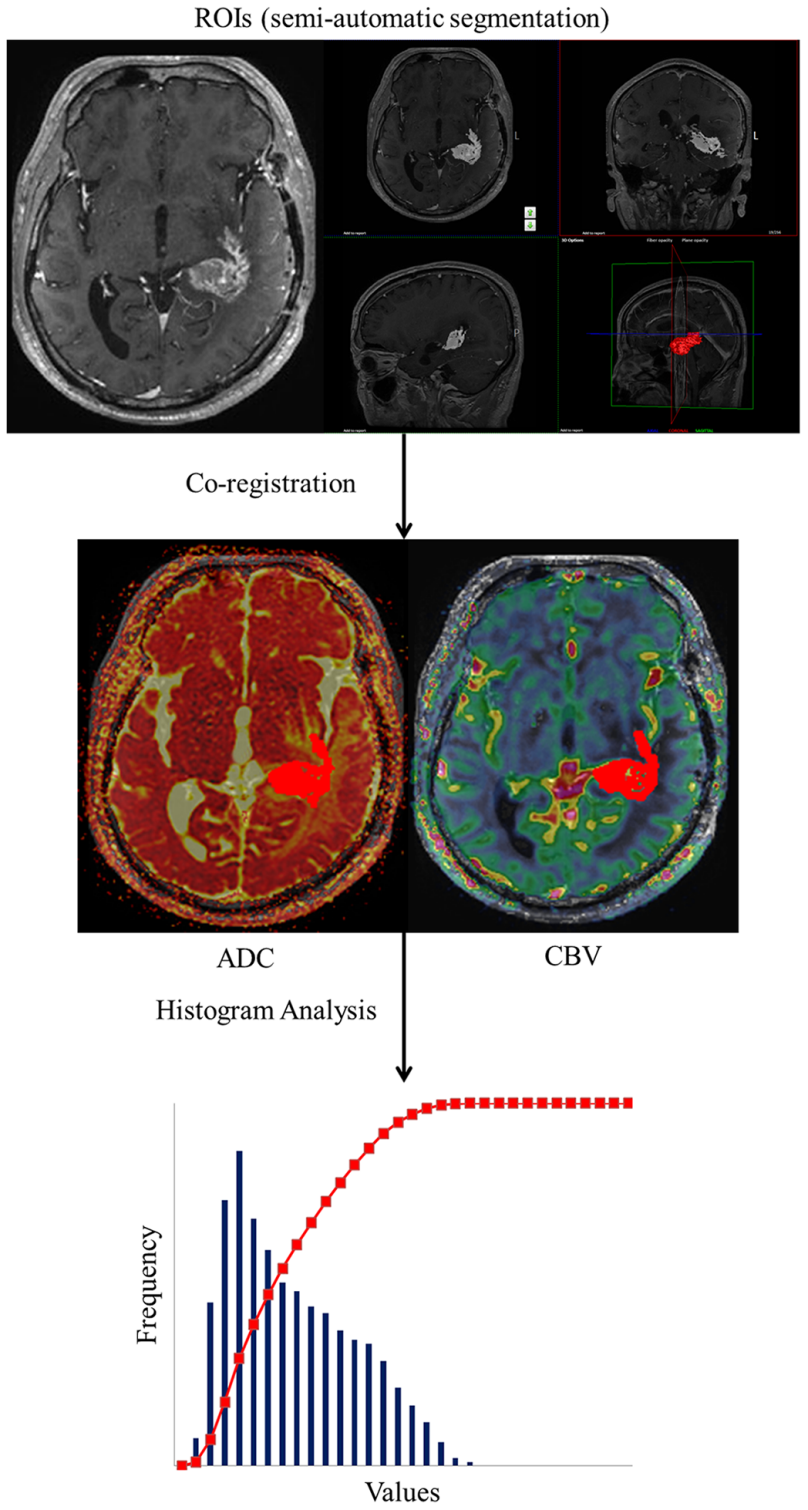
Flow diagram of the histogram analysis. The ROIs were determined with the semiautomatic segmentation method; then, contrast-enhanced T1WI and nCBV or ADC maps were co-registered. Finally, the nCBV and ADC values from the whole pixels of the ROIs were calculated and analyzed with a histogram approach.

Then, one radiologist (J.H.K.; 2 years of brain MRI experience) performed histogram analysis in the manner described below. The nCBV histograms were plotted with the nCBV on the x-axis, with a bin size of 0.2, and the y-axis was expressed as a percentage of the total lesion volume by dividing the frequency in each bin by the total number of analyzed voxels. For further quantitative analysis, cumulative nCBV histograms were obtained from the nCBV histograms, in which the cumulative number of observations in all of the bins up to the specified bin was mapped on the y-axis as percentages. The following parameters were derived from the nCBV histograms: (a) the mean and (b), in the cumulative nCBV histograms, the 99^th^ percentile points (the X^th^ percentile point is the point at which X% of the voxel values that form the histogram are found to the left of the histogram) [27], [29], [30]. The nADC histograms were plotted with the nADC values on the x-axis, with a bin size of 0.1, and the y-axis was expressed as a percentage of the total lesion volume by dividing the frequency in each bin by the total number of analyzed voxels. In the same manner as for the cumulative nCBV histograms, the cumulative nADC histograms were obtained from the nADC histograms. The mean nADC was derived from the nADC histograms. The 5^th^ percentile point of the cumulative nADC histograms was also derived [29], [30] (Fig. 2).

### Statistical Analysis

All statistical analyses were performed with SPSS software, version 21.0 (IBM). All reported *P* values were two-sided; a *P* value of less than 0.05 was considered statistically significant.

The measurable enhancing lesion (+) and (−) group clinical characteristics were compared using Student's t-test for non-categorical variables and Fisher's exact or chi-square tests for categorical variables. Student's t-test was also used to evaluate the difference in the histogram parameters and ROI volumes between the non-progression and progression groups of patients with measurable enhancing lesions. The proportion of patients who experienced the pseudoprogression between the non-progression and progression groups was assessed by Fisher's exact test. Survival curves for the PFS were constructed using the Kaplan-Meier method, and log-rank tests were carried out to evaluate the differences between the two groups, which were divided by the presence of a measurable enhancing lesion or the nCBV cutoff value (4.5). With the simple linear regression model, the ROI volume of the measurable enhancing lesion, 99^th^ percentile nCBV value and 5^th^ percentile nADC value were correlated with the PFS of patients with measurable enhancing lesions.

## Results

### Patient Clinical Characteristics

The characteristics of the patients in the two groups (with or without measurable enhancing lesion) were comparatively well balanced. The mean age of the 59 patients was 50 years, and 57 patients (97%) underwent gross total resection. Two patients, who only received stereotactic biopsy without surgical resection, were divided equally between the two groups. Additionally, the Karnofsky performance scores were more than 70 in 56 patients (95%) at the time of the first follow-up MRI after adjuvant TMZ. MGMT promoter methylation was less in the measurable enhancing lesion (+) group than in the measurable enhancing lesion (−) group, but this difference was not statistically significant (56% vs 71%) (Table 1).

**Table 1:** Patient Clinical Characteristics.

Characteristics	Total	Measurable enhancing lesion (+)	Measurable enhancing lesion (−)	*P* Value
No. of patients	59	27	32	
Age (y)*	50.47±14.87	53.7±12.01	47.75±16.61	.117†
Karnofsky performance score – no. (%)				.588‡
<70	3 (5)	2 (7)	1 (3)	
≧70	56 (95)	25 (93)	31 (97)	
Surgery – no. (%)				1.000‡
Biopsy	2 (3)	1 (4)	1 (3)	
Resection	57 (97)	26 (96)	31 (97)	
Methylated MGMT promoter – no. (%)				.192‡
Negative	21 (36)	12 (44)	9 (29)	
Positive	38 (64)	15 (56)	23 (71)	

Note. – Unless otherwise indicated, data are given as the number of patients.

*Data are the mean±standard deviation.

† Difference between the groups was evaluated with Student's t-test.

‡ Difference between the groups was evaluated with Fisher's exact or chi-square tests.

### PFS according to the Presence of a Measurable Enhancing Lesion

**Figure 3:**
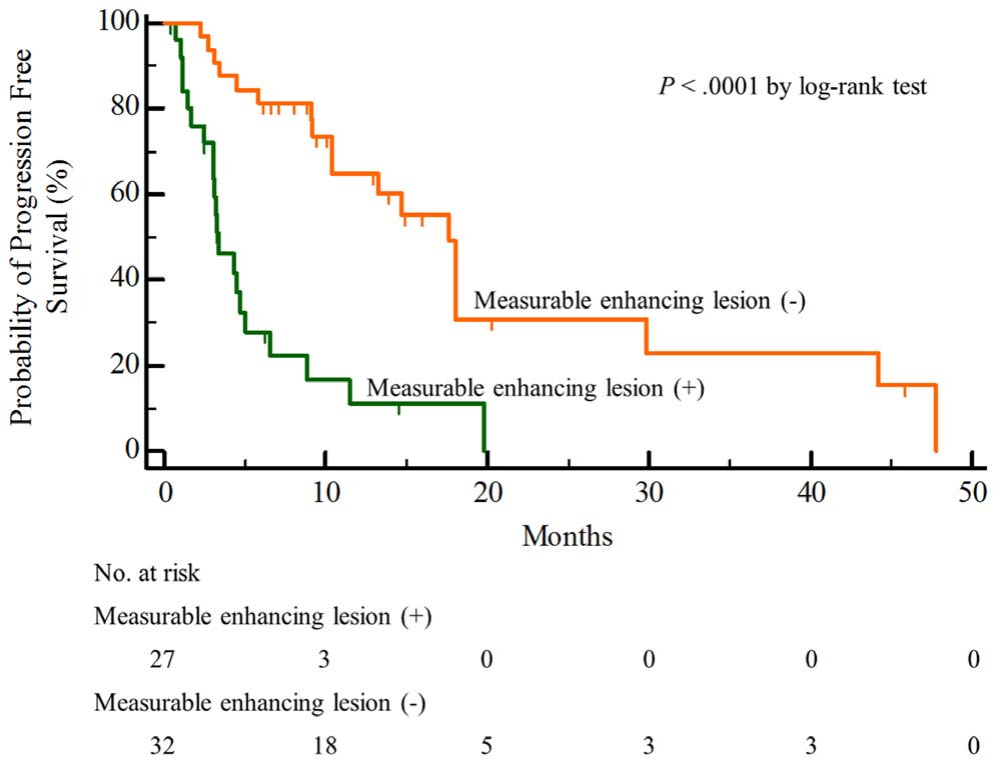
Kaplan-Meier estimates of the progression free survival according to the presence of measurable enhancing lesion.

The median PFS was shorter in patients with measurable enhancing lesions than in those lacking a measurable enhancing lesion, 3.3 months [95% confidence interval (CI), 1.7 to 5.0] vs 17.6 months (95% CI, 11.9 to 23.4), *P*<.0001 by the log-rank test (Table 2) (Fig. 3). Additionally, the one-year PFS rate was lower in patients with measurable enhancing lesions than in those without measurable enhancing lesions, 11.1% (95% CI, 0 to 25.2) vs 60.2% (95% CI, 41.4 to 79.0) (Table 2).

**Table 2:** Progression Free Survival According to the Presence of a Measurable Enhancing Lesion.

Variable	Measurable enhancing lesion (+) (*n* = 27)	Measurable enhancing lesion (−) (*n* = 32)
	*value (95% CI)*	
Median progression free survival (mo)	3.3 (1.7–5.0)	17.6 (11.9–23.4)
Progression free survival (%)		
At 3 months	67.8 (49.4–86.2)	90.6 (80.4–100.8)
At 6 months	22.2 (4.6–39.8)	81.3 (67.8–94.8)
At 9 months	16.6 (0.3–32.9)	77.4 (62.5–92.3)
At 12 months	11.1 (0–25.2)	60.2 (41.4–79.0)

### Histogram Parameters in the Patients with Measurable Enhancing Lesions

The ROI volumes of the measurable enhancing lesion in the non-progression group (mean±standard deviation; 32.1±46.2 cm^3^) were comparable to those in the progression group (20.2±21.3 cm^3^) (*P* = .646). In addition, the proportion of patients who experienced the pseudoprogression did not differ between the two groups (*P* = 1.000 by Fisher's exact test). However, the 99^th^ percentile nCBV value for the non-progression group (6.17±1.57) was significantly higher than for the progression group (4.44±1.45) (*P* = .039). There were no significant differences between the two groups for other histogram parameters (Table 3).

**Table 3:** Histogram Parameters of Patients with Measurable Enhancing Lesions.

	Measurable enhancing lesion (+)		
	Non-progression (*n* = 4)	Progression (*n* = 23)	*P* Value
ROI volume (cm^3^)	32.1±46.2	20.2±21.3	.646
Pseudoprogression – no. (%)*			1.000†
Negative	2 (50)	13 (59)	
Positive	2 (50)	9 (41)	
nADC			
Mean	1.900±0.276	1.936±0.324	.837
Fifth percentile	0.950±0.379	1.142±0.203	.139
nCBV			
Mean	2.00±0.43	1.55±0.61	.172
Ninety-ninth percentile	6.17±1.57	4.44±1.45	.039‡

Note. – Unless otherwise indicated, data are given as the mean±standard deviation and the difference between the groups was evaluated with Student's t-test.

*The pseudoprogression was not available for one patient in the progression group.

† Difference between the groups was evaluated with Fisher's exact test.

‡ Difference between the groups was significant (*P*<.05).

**Figure 4:**
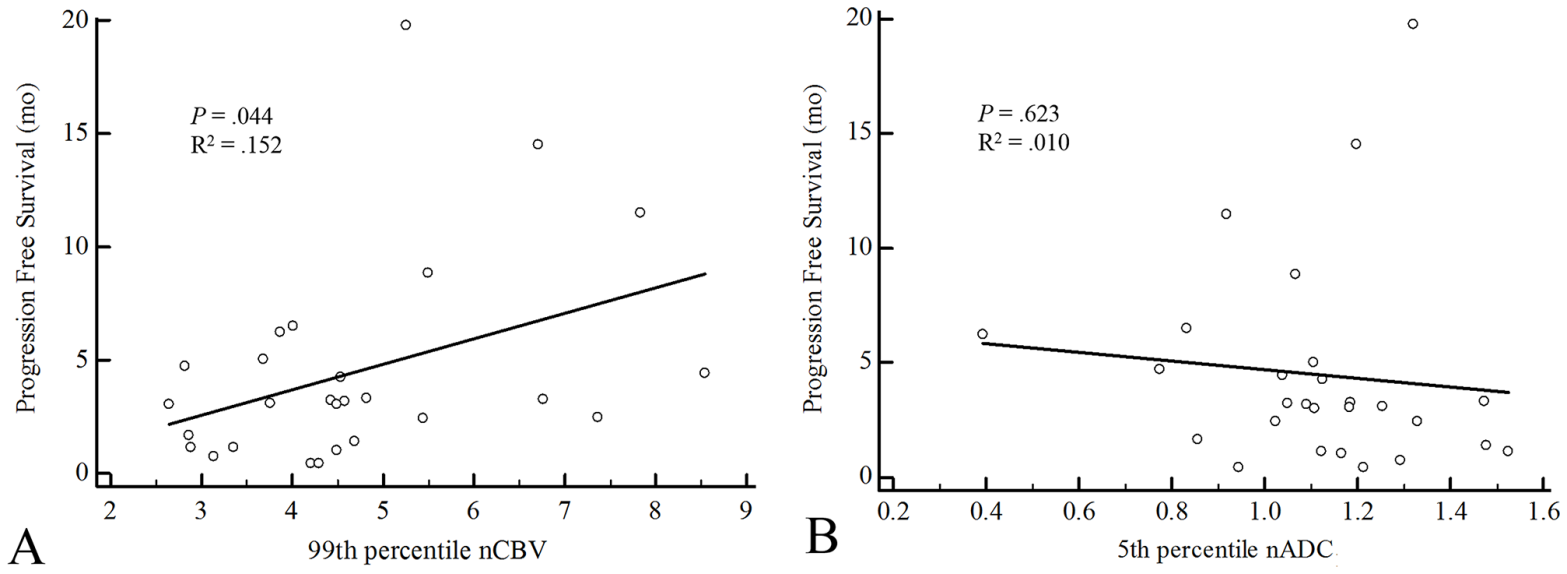
The linear regression plots of the progression free survival against the (A) 99^th^ percentile nCBV (R^2^ = .152, *P* = .044) and (B) 5^th^ percentile nADC values (R^2^ = .010, *P* = .623).

There was a statistically significant positive correlation between the 99^th^ percentile nCBV value of measurable enhancing lesions and the PFS (*P* = .044, R^2^ = .152). However, we could not find a significant correlation between the PFS and 5^th^ percentile nADC value (*P* = .623, R^2^ = .010) (Fig. 4). Furthermore, the ROI volume of the measurable enhancing lesion did not correlate with the PFS (*P* = .290, R^2^ = .045).

### PFS according to the 99^th^ percentile nCBV value in Patients with Measurable Enhancing Lesions

**Figure 5:**
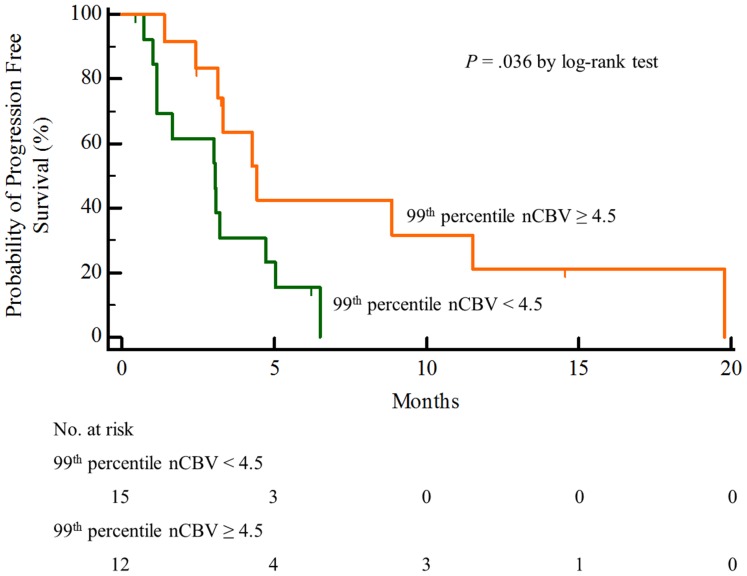
Kaplan-Meier estimates of the progression free survival for patients with measurable enhancing lesions separated by the 99th percentile nCBV (cut off value = 4.5).

The median PFS was higher in the patients with a 99^th^ percentile nCBV value ≧4.5 (*n* = 12) than in those with a 99^th^ percentile nCBV value <4.5 (*n* = 15), 4.4 months (95% CI, 2.8 to 6.0) vs 3.1 months (95% CI, 1.4 to 4.8), *P* = .036 by the log-rank test (Fig. 5).

**Figure 6:**
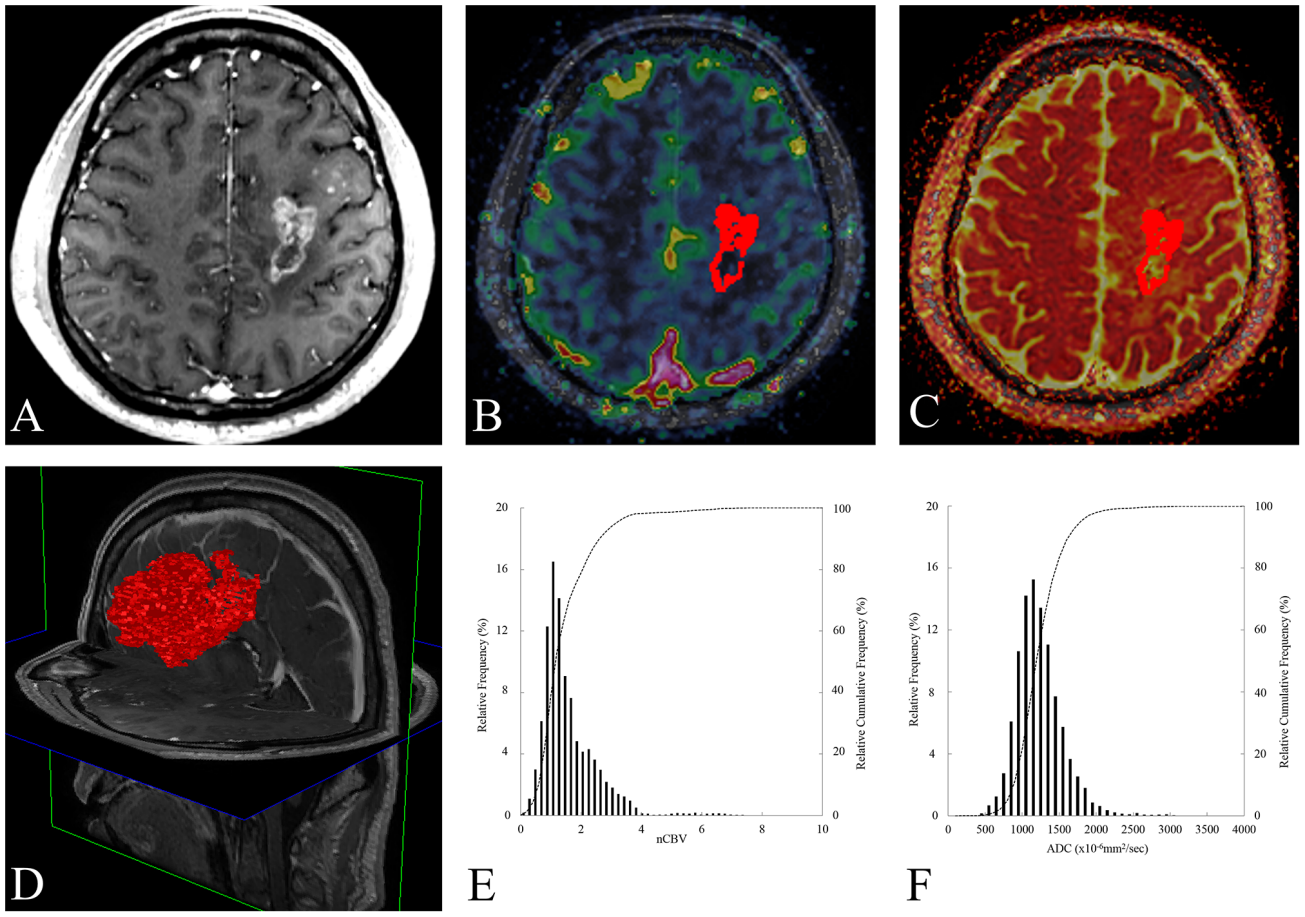
A 56-year-old male glioblastoma patient with a measurable enhancing lesion after adjuvant TMZ. The 99^th^ percentile nCBV value and PFS of this patient were 5.5 and 8.9 months, respectively. (A) An axial contrast-enhanced T1WI obtained within 3 months after CCRT and adjuvant TMZ shows a measurable enhancing lesion in the left frontal cortex. (B) The nCBV map and ROIs (red color) are displayed as a color overlay on the contrast-enhanced T1WI. (C) ADC map with ROIs (red color) are displayed as a color overlay (in hot scale) on the contrast-enhanced T1WI. (D) The volume rendering contrast-enhancement T1WI from measurable enhancing lesion shows the volume (red color) of measurable enhancing lesion at the time of progression. (E, F) The nCBV and ADC histograms of measurable enhancing lesion.

**Figure 7:**
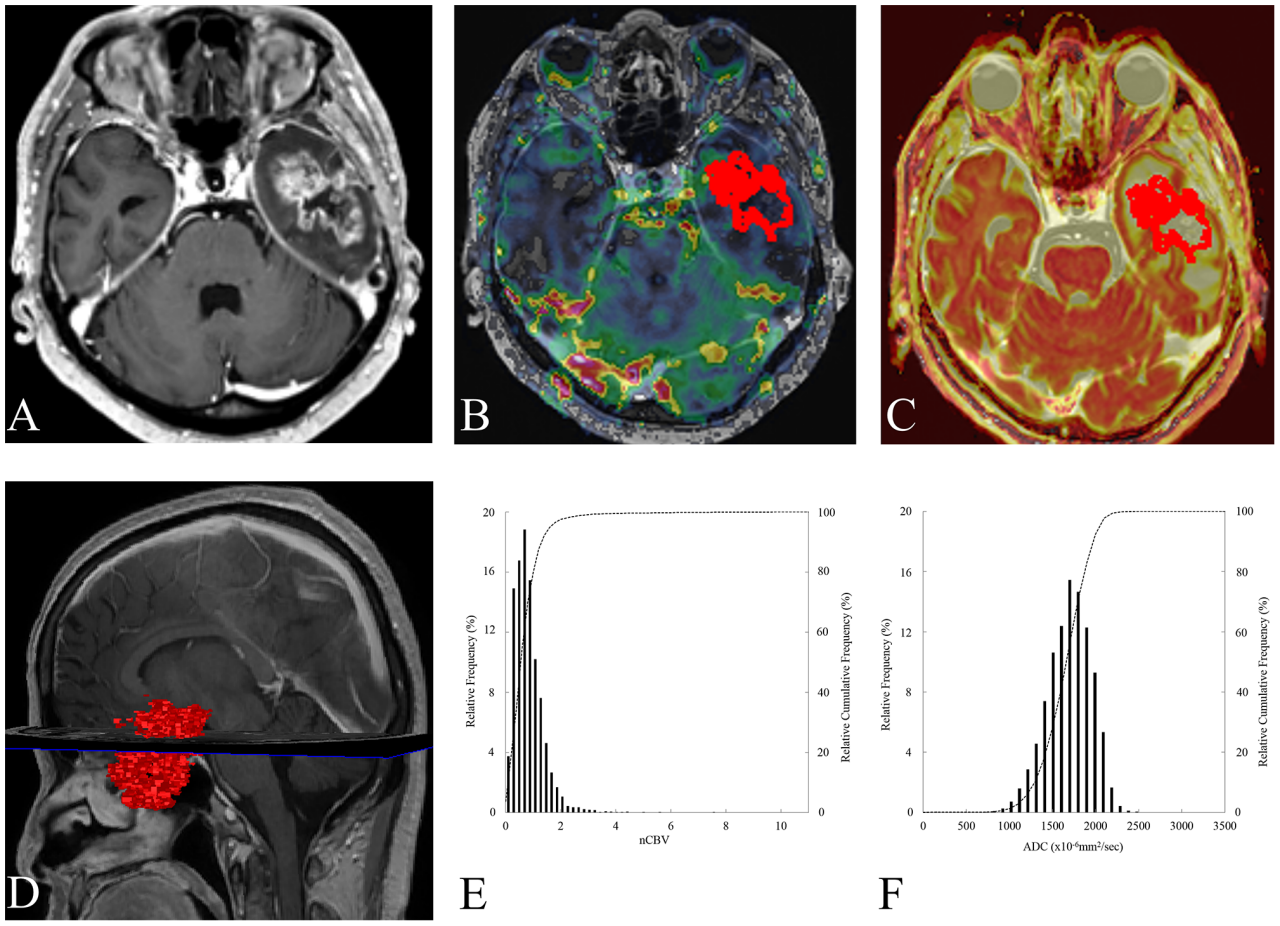
A 60-year-old male glioblastoma patient with a measurable enhancing lesion after adjuvant TMZ. The 99^th^ percentile nCBV value and PFS of this patient were 2.9 and 1.1 months, respectively. (A) An axial contrast-enhanced T1WI obtained within 3 months after CCRT and adjuvant TMZ shows a measurable enhancing lesion in the left temporal lobe. (B) The nCBV map and ROIs (red color) are displayed as a color overlay on the contrast-enhanced T1WI. (C) ADC map with ROIs (red color) are displayed as a color overlay (in hot scale) on the contrast-enhanced T1WI. (D) The volume rendering contrast-enhancement T1WI from measurable enhancing lesion shows the volume (red color) of measurable enhancing lesion at the time of progression. (E, F) The nCBV and ADC histograms of measurable enhancing lesion.

The ADC, nCBV maps and histograms of two representative cases were shown in Figures 6 and 7.

## Discussion

One of the most important results from our study is the higher median PFS in patients lacking measurable enhancing lesions than in the patients with measurable enhancing lesions (17.6 vs 3.3 months). Additionally, the one-year PFS rate was 60.2% in the group lacking measurable enhancing lesions, which was higher than that (11.1%) of the group with measurable enhancing lesions. Several studies have already emphasized that residual tumor after resection is an independent prognostic factor for survival in cases of low- and high-grade gliomas [31]–[33]. Our study suggests that measurable enhancing lesions at the end of standard treatment for GBM (adjuvant TMZ following gross total resection and CCRT with TMZ) could be an important prognostic factor in GBM patients. Therefore, the patients whose follow-up MRIs show a measurable enhancing lesion after adjuvant TMZ might need additional therapy, such as novel chemotherapy and surgical resection, or close observation. Additional clinical studies are needed to evaluate the benefit of additional treatments in patients with measurable enhancing lesions after completing standard treatment for GBM.

In our study results, we found significant positive correlation between the 99^th^ percentile nCBV value of a measurable enhancing lesion and the PFS (*P* = .044, R^2^ = .152). Furthermore, the 99^th^ percentile nCBV value of the non-progression group was significantly higher than that of the progression group in patients with measurable enhancing lesions (6.17±1.57 vs 4.44±1.45, *P* = .039), and the median PFS was significantly higher in the patients with a 99^th^ percentile nCBV value ≧4.5 than in the patients with a 99^th^ percentile nCBV value <4.5 (4.4 months vs 3.1 months, *P* = .036). Our results seem to contradict previous reports. According to several previous studies, increased rCBV or high histogram parameters, such as the peak height position, after CCRT in GBM patients were associated with a poor prognosis [20], [34]. Additionally, a recent study by Kim et al. [21] demonstrated that high 99^th^ percentile nCBV values were helpful for detecting high-grade glioma. Furthermore, the high rCBV or histogram parameters reflect the relative fraction of the high-grade tumor portion compared with treatment-related brain parenchymal changes because the tumor usually comprises a mixture of viable tumor tissue and treatment-induced necrosis [34]. However, the CBV produced by the DSC-PWI technique can provide physiologic information on the tumor vascularity [35]–[37]. Thus, decreased 99^th^ percentile nCBV values are indicative of decreases in vascularity and tissue perfusion, which can induce relatively more hypoxic conditions for tumor cells. In previous studies [38]–[40], hypoxic cancer cells were shown to be more resistant to radiation or cytotoxic drugs as well as more progressive and aggressive. Therefore, the low 99^th^ percentile nCBV values seem to indirectly reflect hypoxic conditions, which could make cancer cells more aggressive and resistant to treatment; thus, low 99^th^ percentile nCBV values for measurable enhancing lesions after standard GBM treatment may be associated with poor prognosis.

In terms of the ADC values, we found no significant correlation between the 5^th^ percentile nADC value and PFS (*P* = .623) as well as no significant differences in the mean and 5^th^ percentile nADC value between the non-progression and progression groups (*P* = .837 and.139, respectively). The ADC values reflect the tumor cellularity; thus, ADC values are higher in cystic or necrotic areas than in the solid component of tumors [41], [42]. In addition, in several previous studies [12], [18], [43], recurrent tumors were shown to have significantly lower ADC values or lower histogram parameters, such as the 5^th^ percentile ADC value, than for radiation injury. We found that measurable enhancing lesions after standard treatment for GBM tend to have similar cellularity regardless of their prognosis, which is likely because enhancing lesions can consist of several microenvironments, such as necrosis, inflammation, and viable tumor cells.

As with any retrospective analysis, this study has inherent biases and other limitations. First, our sample size was relatively small. The statistical power was only 5.6% to evaluate the difference in the mean nADC between the non-progression (*n* = 4) and progression (*n* = 23) groups by using Student's t-test. However, there may actually be no significant difference in the mean nADC between these two groups because the difference in our study was only 0.036 considered clinically meaningless. Furthermore, though there was also a significant difference in the 99^th^ percentile nCBV between these two groups, a sample size of non-progression group was only 4. Therefore, large population studies are required to validate our results. Second, we used multiple MRI scanners with different field strengths (e.g., 1.5 and 3.0 T scanners) from different manufacturers, and the scan parameters were slightly different for each machine. Although we normalized the CBV and ADC values to minimize the effects of the different magnetic field strengths and type of MRI scanners, there could be a slight bias in the image analysis of the ADC and nCBV maps. Third, our survival analysis data censoring rate was relatively high, which could decrease the reliability of the Kaplan-Meier analysis. To verify our findings, further studies with less censoring of the data should be performed.

In conclusion, the presence of an enhancing lesion seems to be an important factor for predicting the PFS in GBM patients who have received standard treatments including CCRT with TMZ and adjuvant TMZ following surgery. Additionally, an increase in the 99^th^ percentile nCBV values of measurable enhancing lesions has a good correlation with improved PFS.

## References

[1] Omuro A, DeAngelis LM (2013) Glioblastoma and other malignant gliomas: a clinical review. JAMA 310:1842–1850. 24193082 10.1001/jama.2013.280319

[2] Jeon HJ, Kong DS, Park KB, Lee JI, Park K, et al (2009) Clinical outcome of concomitant chemoradiotherapy followed by adjuvant temozolomide therapy for glioblastaomas: single-center experience. Clin Neurol Neurosurg 111:679–682. 19640635 10.1016/j.clineuro.2009.06.013

[3] Quinn JA, Jiang SX, Reardon DA, Desjardins A, Vredenburgh JJ, et al (2009) Phase II trial of temozolomide (TMZ) plus irinotecan (CPT-11) in adults with newly diagnosed glioblastoma multiforme before radiotherapy. J Neurooncol 95:393–400. 19533023 10.1007/s11060-009-9937-x PMC2835159

[4] Johnson DR, Leeper HE, Uhm JH (2013) Glioblastoma survival in the United States improved after Food and Drug Administration approval of bevacizumab: a population-based analysis. Cancer 119:3489–3495. 23868553 10.1002/cncr.28259

[5] Stupp R, Mason WP, van den Bent MJ, Weller M, Fisher B, et al (2005) Radiotherapy plus concomitant and adjuvant temozolomide for glioblastoma. N Engl J Med 352:987–996. 15758009 10.1056/NEJMoa043330

[6] Stupp R, Hegi ME, Mason WP, van den Bent MJ, Taphoorn MJ, et al (2009) Effects of radiotherapy with concomitant and adjuvant temozolomide versus radiotherapy alone on survival in glioblastoma in a randomised phase III study: 5-year analysis of the EORTC-NCIC trial. Lancet Oncol 10:459–466. 19269895 10.1016/S1470-2045(09)70025-7

[7] Mirimanoff RO, Gorlia T, Mason W, Van den Bent MJ, Kortmann RD, et al (2006) Radiotherapy and temozolomide for newly diagnosed glioblastoma: recursive partitioning analysis of the EORTC 26981/22981-NCIC CE3 phase III randomized trial. J Clin Oncol 24:2563–2569. 16735709 10.1200/JCO.2005.04.5963

[8] Macdonald DR, Cascino TL, Schold SC Jr, Cairncross JG (1990) Response criteria for phase II studies of supratentorial malignant glioma. J Clin Oncol 8:1277–1280. 2358840 10.1200/JCO.1990.8.7.1277

[9] Wen PY, Macdonald DR, Reardon DA, Cloughesy TF, Sorensen AG, et al (2010) Updated response assessment criteria for high-grade gliomas: response assessment in neuro-oncology working group. J Clin Oncol 28:1963–1972. 20231676 10.1200/JCO.2009.26.3541

[10] Gahramanov S, Muldoon LL, Varallyay CG, Li X, Kraemer DF, et al (2013) Pseudoprogression of glioblastoma after chemo- and radiation therapy: diagnosis by using dynamic susceptibility-weighted contrast-enhanced perfusion MR imaging with ferumoxytol versus gadoteridol and correlation with survival. Radiology 266:842–852. 23204544 10.1148/radiol.12111472 PMC3579176

[11] Hein PA, Eskey CJ, Dunn JF, Hug EB (2004) Diffusion-weighted imaging in the follow-up of treated high-grade gliomas: tumor recurrence versus radiation injury. AJNR Am J Neuroradiol 25:201–209. 14970018 PMC7974622

[12] Asao C, Korogi Y, Kitajima M, Hirai T, Baba Y, et al (2005) Diffusion-weighted imaging of radiation-induced brain injury for differentiation from tumor recurrence. AJNR Am J Neuroradiol 26:1455–1460. 15956515 PMC8149095

[13] Barajas RF Jr, Chang JS, Segal MR, Parsa AT, McDermott MW, et al (2009) Differentiation of recurrent glioblastoma multiforme from radiation necrosis after external beam radiation therapy with dynamic susceptibility-weighted contrast-enhanced perfusion MR imaging. Radiology 253:486–496. 19789240 10.1148/radiol.2532090007 PMC2770116

[14] Hu LS, Baxter LC, Smith KA, Feuerstein BG, Karis JP, et al (2009) Relative cerebral blood volume values to differentiate high-grade glioma recurrence from posttreatment radiation effect: direct correlation between image-guided tissue histopathology and localized dynamic susceptibility-weighted contrast-enhanced perfusion MR imaging measurements. AJNR Am J Neuroradiol 30:552–558. 19056837 10.3174/ajnr.A1377 PMC7051449

[15] Hilario A, Ramos A, Perez-Nunez A, Salvador E, Millan JM, et al (2012) The added value of apparent diffusion coefficient to cerebral blood volume in the preoperative grading of diffuse gliomas. AJNR Am J Neuroradiol 33:701–707. 22207304 10.3174/ajnr.A2846 PMC8050428

[16] Arvinda HR, Kesavadas C, Sarma PS, Thomas B, Radhakrishnan VV, et al (2009) Glioma grading: sensitivity, specificity, positive and negative predictive values of diffusion and perfusion imaging. J Neurooncol 94:87–96. 19229590 10.1007/s11060-009-9807-6

[17] Song YS, Choi SH, Park CK, Yi KS, Lee WJ, et al (2013) True progression versus pseudoprogression in the treatment of glioblastomas: a comparison study of normalized cerebral blood volume and apparent diffusion coefficient by histogram analysis. Korean J Radiol 14:662–672. 23901325 10.3348/kjr.2013.14.4.662 PMC3725362

[18] Chu HH, Choi SH, Ryoo I, Kim SC, Yeom JA, et al (2013) Differentiation of True Progression from Pseudoprogression in Glioblastoma Treated with Radiation Therapy and Concomitant Temozolomide: Comparison Study of Standard and High-b-Value Diffusion-weighted Imaging. Radiology 269:831–840. 23771912 10.1148/radiol.13122024

[19] Suh CH, Kim HS, Choi YJ, Kim N, Kim SJ (2013) Prediction of Pseudoprogression in Patients with Glioblastomas Using the Initial and Final Area Under the Curves Ratio Derived from Dynamic Contrast-Enhanced T1-Weighted Perfusion MR Imaging. AJNR Am J Neuroradiol 34:2278–86. 23828115 10.3174/ajnr.A3634 PMC7965196

[20] Kim HS, Kim JH, Kim SH, Cho KG, Kim SY (2010) Posttreatment high-grade glioma: usefulness of peak height position with semiquantitative MR perfusion histogram analysis in an entire contrast-enhanced lesion for predicting volume fraction of recurrence. Radiology 256:906–915. 20634429 10.1148/radiol.10091461

[21] Kim H, Choi SH, Kim JH, Ryoo I, Kim SC, et al (2013) Gliomas: application of cumulative histogram analysis of normalized cerebral blood volume on 3 T MRI to tumor grading. PLoS One 8:e63462. 23704910 10.1371/journal.pone.0063462 PMC3660395

[22] Rosen BR, Belliveau JW, Vevea JM, Brady TJ (1990) Perfusion imaging with NMR contrast agents. Magn Reson Med 14:249–265. 2345506 10.1002/mrm.1910140211

[23] Ostergaard L, Weisskoff RM, Chesler DA, Gyldensted C, Rosen BR (1996) High resolution measurement of cerebral blood flow using intravascular tracer bolus passages. Part I: Mathematical approach and statistical analysis. Magn Reson Med 36:715–725. 8916022 10.1002/mrm.1910360510

[24] Boxerman JL, Schmainda KM, Weisskoff RM (2006) Relative cerebral blood volume maps corrected for contrast agent extravasation significantly correlate with glioma tumor grade, whereas uncorrected maps do not. AJNR Am J Neuroradiol 27:859–867. 16611779 PMC8134002

[25] Wetzel SG, Cha S, Johnson G, Lee P, Law M, et al (2002) Relative cerebral blood volume measurements in intracranial mass lesions: interobserver and intraobserver reproducibility study. Radiology 224:797–803. 12202717 10.1148/radiol.2243011014

[26] Bjornerud A (2003) The ICE software package: direct co-registration of anatomical and functional datasets using DICOM image geometry information. Proc Hum Brain Mapping 19:1018.

[27] Jung SC, Choi SH, Yeom JA, Kim JH, Ryoo I, et al (2013) Cerebral blood volume analysis in glioblastomas using dynamic susceptibility contrast-enhanced perfusion MRI: a comparison of manual and semiautomatic segmentation methods. PLoS One 8:e69323. 23950891 10.1371/journal.pone.0069323 PMC3738566

[28] Hwang EJ, Cha Y, Lee AL, Yun TJ, Kim TM, et al (2013) Early response evaluation for recurrent high grade gliomas treated with bevacizumab: a volumetric analysis using diffusion-weighted imaging. J Neurooncol 112:427–435. 23417358 10.1007/s11060-013-1072-z

[29] Kang Y, Choi SH, Kim YJ, Kim KG, Sohn CH, et al (2011) Gliomas: Histogram analysis of apparent diffusion coefficient maps with standard- or high-b-value diffusion-weighted MR imaging–correlation with tumor grade. Radiology 261:882–890. 21969667 10.1148/radiol.11110686

[30] Tozer DJ, Jager HR, Danchaivijitr N, Benton CE, Tofts PS, et al (2007) Apparent diffusion coefficient histograms may predict low-grade glioma subtype. NMR Biomed 20:49–57. 16986106 10.1002/nbm.1091

[31] McGirt MJ, Chaichana KL, Attenello FJ, Weingart JD, Than K, et al. (2008) Extent of surgical resection is independently associated with survival in patients with hemispheric infiltrating low-grade gliomas. Neurosurgery 63: 700–707; author reply 707–708. 10.1227/01.NEU.0000325729.41085.73 18981880

[32] Stummer W, Reulen HJ, Meinel T, Pichlmeier U, Schumacher W, et al (2008) Extent of resection and survival in glioblastoma multiforme: identification of and adjustment for bias. Neurosurgery 62:564–576 discussion 564–576. 18425006 10.1227/01.neu.0000317304.31579.17

[33] Sanai N, Berger MS (2008) Glioma extent of resection and its impact on patient outcome. Neurosurgery 62:753–764 discussion 264–756. 18496181 10.1227/01.neu.0000318159.21731.cf

[34] Mangla R, Singh G, Ziegelitz D, Milano MT, Korones DN, et al (2010) Changes in relative cerebral blood volume 1 month after radiation-temozolomide therapy can help predict overall survival in patients with glioblastoma. Radiology 256:575–584. 20529987 10.1148/radiol.10091440

[35] Cha S (2003) Perfusion MR imaging: basic principles and clinical applications. Magn Reson Imaging Clin N Am 11:403–413. 14768726 10.1016/s1064-9689(03)00066-7

[36] Law M, Yang S, Wang H, Babb JS, Johnson G, et al (2003) Glioma grading: sensitivity, specificity, and predictive values of perfusion MR imaging and proton MR spectroscopic imaging compared with conventional MR imaging. AJNR Am J Neuroradiol 24:1989–1998. 14625221 PMC8148904

[37] Law M, Yang S, Babb JS, Knopp EA, Golfinos JG, et al (2004) Comparison of cerebral blood volume and vascular permeability from dynamic susceptibility contrast-enhanced perfusion MR imaging with glioma grade. AJNR Am J Neuroradiol 25:746–755. 15140713 PMC7974484

[38] Amberger-Murphy V (2009) Hypoxia helps glioma to fight therapy. Curr Cancer Drug Targets 9:381–390. 19442057 10.2174/156800909788166637

[39] Brat DJ, Mapstone TB (2003) Malignant glioma physiology: cellular response to hypoxia and its role in tumor progression. Ann Intern Med 138:659–668. 12693889 10.7326/0003-4819-138-8-200304150-00014

[40] Huang LE, Bindra RS, Glazer PM, Harris AL (2007) Hypoxia-induced genetic instability–a calculated mechanism underlying tumor progression. J Mol Med (Berl) 85:139–148. 17180667 10.1007/s00109-006-0133-6

[41] Sugahara T, Korogi Y, Kochi M, Ikushima I, Shigematu Y, et al (1999) Usefulness of diffusion-weighted MRI with echo-planar technique in the evaluation of cellularity in gliomas. J Magn Reson Imaging 9:53–60. 10030650 10.1002/(sici)1522-2586(199901)9:1<53::aid-jmri7>3.0.co;2-2

[42] Padhani AR, Liu G, Koh DM, Chenevert TL, Thoeny HC, et al (2009) Diffusion-weighted magnetic resonance imaging as a cancer biomarker: consensus and recommendations. Neoplasia 11:102–125. 19186405 10.1593/neo.81328 PMC2631136

[43] Lee WJ, Choi SH, Park CK, Yi KS, Kim TM, et al (2012) Diffusion-weighted MR imaging for the differentiation of true progression from pseudoprogression following concomitant radiotherapy with temozolomide in patients with newly diagnosed high-grade gliomas. Acad Radiol 19:1353–1361. 22884399 10.1016/j.acra.2012.06.011

